# The integration of ortho-plastic limb salvage teams in the humanitarian response to violence-related open tibial fractures: evaluating outcomes in the Gaza Strip

**DOI:** 10.1186/s13031-024-00596-3

**Published:** 2024-04-24

**Authors:** Theresa Farhat, Krystel Moussally, Hasan Nahouli, Shahd Abu Hamad, Khulood Abul Qaraya, Zahi Abdul-Sater, Walaa G. El Sheikh, Nadine Jawad, Khouloud Al Sedawi, Mohammed Obaid, Hafez AbuKhoussa, Innocent Nyaruhirira, Hani Tamim, Shehan Hettiaratchy, Anthony M. J. Bull, Ghassan Abu-Sittah

**Affiliations:** 1 Global Health Institute, American University of Beirut, Gefinor Center Block D, 3rd floor, P.O. Box 11-0236, Riad El Solh, Beirut, 1107-2020 Lebanon; 2 Médecins Sans Frontières, Lebanon Branch Office, Middle East Medical Unit, Beirut, Lebanon; 3 Division of Orthopedic Surgery, Department of Surgery, American University of Beirut Medical Center, Beirut, Lebanon; 4 Clinical Research Institute, American University of Beirut Medical Center, Beirut, Lebanon; 5 College of Medicine, Alfaisal University, Riyadh, Saudi Arabia; 6 Operational Centre Brussels, Medical Department, Médecins Sans Frontières, Brussels, Belgium; 7 Operational Centre Brussels, Gaza mission, Médecins Sans Frontières, Gaza, Palestine; 8 Centre for Blast Injury Studies, Imperial College London, London, UK; 9 Department of Surgery and Cancer, Imperial College London, London, UK; 10 Imperial College Healthcare NHS Trust, St Mary’s Hospital, London, UK

**Keywords:** Limb salvage, Open tibial fracture, Lower extremity, War, Conflict, Gunshot wounds, Ortho-plastic, Gaza, Palestine

## Abstract

**Background:**

Limb salvage by ortho-plastic teams is the standard protocol for treating open tibial fractures in high-income countries, but there’s limited research on this in conflict settings like the Gaza Strip. This study assessed the clinical impact of gunshot-related open tibial fractures, compared patient management by orthopedic and ortho-plastic teams, and identified the risk factors for bone non-union in this context.

**Methods:**

A retrospective review of medical records was conducted on Gaza Strip patients with gunshot-induced-open tibial fractures from March 2018 to October 2020. Data included patient demographics, treatments, and outcomes, with at least one year of follow-up. Primary outcomes were union, non-union, infection, and amputation.

**Results:**

The study included 244 injured individuals, predominantly young adult males (99.2%) with nearly half (48.9%) having Gustilo-Anderson type IIIB fractures and more than half (66.8%) with over 1 cm of bone loss. Most patients required surgery, including rotational flaps and bone grafts with a median of 3 admissions and 9 surgeries. Ortho-plastic teams managed more severe muscle and skin injuries, cases with bone loss > 1 cm, and performed less debridement compared to other groups, though these differences were not statistically significant. Non-union occurred in 53% of the cases, with the ortho-plastic team having the highest rate at 63.6%. Infection rates were high (92.5%), but no significant differences in bone or infection outcomes were observed among the different groups. Logistic regression analysis identified bone loss > 1 cm, vascular injury, and the use of a definitive fixator at the first application as predictors of non-union.

**Conclusions:**

This study highlights the severity and complexity of such injuries, emphasizing their significant impact on patients and the healthcare system. Ortho-plastic teams appeared to play a crucial role in managing severe cases. However, further research is still needed to enhance our understanding of how to effectively manage these injuries.

**Supplementary Information:**

The online version contains supplementary material available at 10.1186/s13031-024-00596-3.

## Background

Violence-related injuries continue to be a long-standing public health crisis, with conflict zones like the Gaza Strip being significantly impacted [1, 2]. The endemic nature of conflict in these areas often leads to an overwhelmed and under-resourced healthcare system, adversely affecting the treatment and management of trauma, such as limb injuries [2]. The 2018–2019 Great March of Return (GMR) demonstrations in Gaza, in which almost 200 people were killed and over 28,900 were injured, highlighted the devastating impact of violence-related trauma on the Palestinian population. Of the casualties during GMR, 41% suffered gunshot wounds (GSW) to the lower limb, the most prevalent injury being open tibial fractures [3].

Managing these lower-limb injuries often caused by high-energy mechanisms like gunshots, is challenging. These injuries necessitate multiple surgical procedures and extensive recovery periods, often stretching up to two years. Even with optimal treatment, recovery can be prolonged and with complications [4]. The context of conflict zones, such as the Gaza Strip, exacerbates these challenges. Already under-resourced and overburdened healthcare systems struggle to provide comprehensive treatment to patients with such severe injuries. The scarcity of resources, lack of infrastructure, sporadic electricity, and depleted medical supplies put significant constraints on the ability to deliver adequate care, particularly in emergency situations [5, 6]. Consequently, many salvageable limbs are inevitably amputated, a choice that is frequently opted for in such settings. Compounding these issues is the high risk of complications associated with gunshot-induced open fractures. In addition to osteomyelitis, patients are at an increased risk of wound infections, non-unions, and long-term disability [4]. These risks pose additional burdens on the patient and the healthcare system, further straining the already limited resources. The high frequency of such injuries, particularly during periods of intense conflict like the GMR demonstrations, can lead to a healthcare crisis, with the demand for care far exceeding the capacity of the healthcare system [2].

High-energy injuries like GSW often present significant reconstructive challenges, requiring a collaborative orthopedic and plastic surgery strategy, referred to as the ortho-plastic approach [7]. In this approach, the orthopedic team focuses on managing the initial trauma and stabilizing the fracture, while the plastic team deals with the soft tissue aspects of the injury, including debridement, wound closure, and, when necessary, skin grafting, or tissue transfer to cover the bones [8]. In high-income countries, the integration of ortho-plastic teams from the outset of management has been recognized as a pathway to improved care and functional outcomes [9, 10]. However, in conflict zones and humanitarian settings, particularly in under-resourced countries, the scarcity of plastic surgeons often results in a reliance on orthopedic surgeons alone for the clinical management of war injuries. This lack of specialized ortho-plastic intervention has been associated with increased rates of complication, revision surgeries, infections, and amputation rates [11, 12].

Despite the significant public health implications of violence-related limb injuries in conflict zones, there is a paucity of research focusing on the role of ortho-plastic teams in their management. Most existing data are based on studies conducted on military personnel or in high-income countries where resources are more readily available [13, 14]. Furthermore, the unique challenges of conflict zones, such as the Gaza Strip, highlight the urgent need for building evidence out of these contexts.

In this study, we aimed to assess the clinical burden of gunshot-induced open tibial fractures and compare clinical characteristics and bone outcomes of injured patients managed by orthopedic and ortho-plastic teams during the GMR demonstrations. We also identified the risk factors associated with bone non-union in this context.

## Methods

### Study design and population

This was a retrospective cohort study of patients with at least one open tibial fracture wound secondary to a gunshot sustained during the GMR demonstrations, admitted between 11 March 2018 and 31 October 2019 to at least one of three hospitals in Gaza: Al-Awda, Al-Shifa, and Nasser hospitals, and followed-up until 31 October 2021. Patients admitted with a non-tibial wound, a closed tibial fracture, or an open tibial fracture not resulting from a GSW, or patients who at admission had a previously injured limb, a primary amputation, a polytrauma (multi-system trauma), as well as those admitted to the intensive care unit, and those with early introduction of internal fixations were excluded from the study as it is not recommended in highly contaminated fractures that result from high velocity weapons injuries [15].

Patients were categorized into three different groups based on the treating teams that provided care: (1) “Orthopedic” group including patients who were treated by an orthopedic surgeon only during their case management; (2) “Orthopedic and Plastic” group including patients who were treated by an orthopedic surgeon and a plastic surgeon separately and not as a fully integrated health team at some point in time during their surgical follow-up; and (3) “Ortho-plastic” group including patients who were treated by an integrated ortho-plastic team at some point during their clinical management.

### Study setting

Gaza, part of the occupied Palestinian territories, known as “an open prison” is the third most populated area in the world with around 80% of its population being refugees [16, 17]. It has been under blockade since 2006 and has been experiencing waves of acute conflicts for decades now. This has resulted in destruction of its infrastructure and healthcare system, deteriorating its population health and undermining their living conditions [18]. Since 30 March 2018, Palestinians have been demonstrating, as part of the “Great March of Return” and the 70th Nakba anniversary, for their right of return and the end of the Israeli blockade [6].

It is believed that patients in need of medical care in Gaza shuffle between different facilities to get their relevant medical needs including Al-Awda, Al-Shifa, and Nasser hospitals. Al-Awda hospital is a non-governmental hospital in the north of the Gaza Strip. It represents the largest health facility for the Union of Health Work Committees (UHWC). Médecins Sans Frontières (MSF) has been partnering with Al-Awda hospital since 11 May 2018 where the provision of ortho-plastic and limb reconstructive surgery takes place in the MSF-run part of the hospital. During the GMR, Al-Awda admitted patients in need of reconstructive surgery after they had been stabilized. Those patients would have often undergone their first debridement elsewhere. The two other hospitals included in this study, Shifa and Nasser, are Ministry of Health hospitals. The Shifa medical center is the largest hospital in the Gaza strip; it covers Gaza city and the middle area and is a referral center. It has strong plastic and orthopedic surgery teams that work closely but they are not fully integrated into an ortho-plastic surgical team. Nasser hospital is one of two hospitals covering the south of Gaza. It has no plastic surgery team on site potentially leading to delays in referral and subsequent soft tissue closure after bone fixation. These hospitals represent the main catchment areas for treating patients with GSW by virtue of proximity to clash points and Ministry of Health (MoH) Trauma Stabilization Points [19, 20].

### Data collection and variables

Deidentified study data was retrospectively retrieved from routine electronic medical records when available on site (Al-Awda hospital) and from patients’ medical records when no other database was available or to complete data that was missing in electronic records. Other data sources used was the database run by the Medical Aid for Palestinians (MAP)-UK for the limb salvage unit at Al-Shifa hospital. Variables collected included patients’ socio-demographics, comorbidities, injury characteristics, clinical and surgical data, and outcomes (including union, non-union, wound healing, infection, and amputation). In addition, hospital variables such as date of admission, date of discharge, readmission and length of hospital stay were collected.

### Main outcome measures

The primary outcome measures were bone union and non-union. Bone non-union was defined as a non-healed fracture confirmed by radiographic imaging at least 12 months from the date of injury and by clinical assessment and X-rays in line with the approach of treating surgeons in Gaza. Union was defined as a bone that completely healed or healed abnormally (mal union). Secondary outcomes reported were wound healing, infection, and delayed amputation when they were recorded in patients’ records at any point in time within the study period. Delayed amputation was defined as an amputation occurring more than 30 days post-injury. Wound healing and infection did not follow standard common definitions but were rather reported as in patients’ records following the clinical assessment of the corresponding healthcare treating teams in the field. Multiple outcomes were recorded per patient.

### Statistical analysis

Descriptive analysis was performed. Categorical variables were presented using counts and proportions, while continuous variables were presented using mean with standard deviation (SD) or median with interquartile range (IQR). Clinical characteristics and outcomes were compared between the different surgical teams using chi-square test, Fisher’s exact test, or one-way ANOVA as appropriate. Similarly, characteristics were compared between patients with union and non-union outcomes. Variables with a *p*-value < 0.20 on the univariate analysis were entered in a stepwise multivariable logistic regression model to identify risk factors for non-union. The treating team variable was imposed in the model regardless of its level of significance, and variables deemed clinically significant were also included. Variables with missing values > 5% were excluded from this analysis. A sensitivity analysis was also conducted for variables with < 5% of missing values. They were replaced by the means for continuous variables and distributed across categories proportionally to the distribution in the rest of the sample for categorical variables. Results were presented as odds ratio (OR) and 95% confidence interval (CI). Significance was set at a *p*-value < 0.05. Data analysis was done using IBM SPSS version 28 (IBM, New York, NY, USA).

### Ethical approval

The research protocol was approved by the Helsinki Committee for Research Ethics in the Palestinian Ministry of Health in Gaza and the health authorities (PHRC/HC/788/20). The study fulfilled the exemption criteria set by MSF Ethics Review Board (ERB) (Geneva Switzerland) for a posteriori analysis of routinely collected clinical data and thus did not require full review (ID 20,101). In addition, the protocol was approved by the Institutional Review Board (IRB) at the American University of Beirut in Lebanon (BIO-2020-0508).

### Patient involvement

No patients were involved in the conceptualisation or conduct of this study due to the nature of the study as a retrospective study.

## Results

### Overview of patient injury and surgical characteristics

The study comprised 244 patients who met the eligibility criteria, with the majority being male (99.2%, *n* = 242/244) and young adults, with a mean age of 28.8 years (SD ± 8.3) (Table 1). Most patients (98.8%, *n* = 241/244) were admitted to the hospital on the same day as their injury. Mid-shaft fractures (42.0%, *n* = 102/243), nerve injuries (74.8%, *n* = 178/238), and large skin injuries (95.8%, *n* = 230/240) were among the most common injuries observed. Nearly half of the fractures (48.9%, *n* = 116/237) were classified as Gustilo-Anderson type IIIB, and more than half had over 1 cm of bone loss (66.8%, *n* = 161/241) (Table 2).

**Table 1:** Demographic characteristics of study patients, 11 May 2018 to 31 October 2020, Gaza, Palestine

Characteristics	All Patients (*N* = 244)	Orthopedic (*N* = 64)	Orthopedic and Plastic (*N* = 135)	Ortho-Plastic (*N* = 45)	*p*-value
**Socio-demographic**					
**Age, years** – Mean ± SD	28.8 ± 8.3	29.9 ± 9.2	27.7 ± 7.5	30.8 ± 8.6	0.05*
**Gender** – n (%)					
Male	242 (99.2%)	63 (98.4%)	134 (99.3%)	45 (100.0%)	0.69‡
Female	2 (0.8%)	1 (1.6%)	1 (0.7%)	0 (0.0%)	
**Smoking** – n (%)					
Yes	114 (48.1%)	29 (46.8%)	65 (48.9%)	21 (48.8%)	0.96†
No	123 (51.9%)	33 (53.2%)	68 (51.1%)	22 (51.2%)	

*One-Way ANOVA

†Pearson’s Chi-Square Test

‡Fisher’s Exact Test

[All % are calculated out of the available data]

**Table 2:** Clinical injury characteristics of study patients, 11 May 2018 to 31 October 2020, Gaza, Palestine

Characteristics	All Patients (*N* = 244)	Orthopedic (*N* = 64)	Orthopedic and Plastic (*N* = 135)	Ortho-Plastic (*N* = 45)	*p*-value
**Clinical and injury - at presentation**					
**Site of injury** – n (%)					
Proximal	71 (29.2%)	20 (31.7%)	37 (27.4%)	14 (31.1%)	0.67†
Mid	102 (42.0%)	26 (41.3%)	61 (45.2%)	15 (33.3%)	
Distal	70 (28.8%)	17 (27.0%)	37 (27.4%)	16 (35.6%)	
**Fibula involved** – n (%)					
Yes	133 (54.7%)	32 (50.8%)	71 (52.6%)	30 (66.7%)	0.20†
No	110 (45.3%)	31 (49.2%)	64 (47.4%)	15 (33.3%)	
**Nerve Injury** – n (%)					
Yes	178 (74.8%)	42 (68.9%)	99 (74.4%)	37 (84.1%)	0.21†
No	60 (25.2%)	19 (31.1%)	34 (25.6%)	7 (15.9%)	
**Vessel Injury** – n (%)					
Artery	97 (40.4%)	25 (41.0%)	52 (38.8%)	20 (44.4%)	0.80†
No	143 (59.6%)	36 (59.0%)	82 (61.2%)	25 (55.6%)	
Vein	52 (21.7%)	14 (23.0%)	24 (17.9%)	14 (31.1%)	0.17†
No	188 (78.3%)	47 (77.0%)	110 (82.1%)	31 (68.9%)	
**Muscle Injury** – n (%)					
Mild	67 (27.9%)	30 (48.4%)	31 (23.1%)	6 (13.6%)	**< 0.001**†
Moderate	144 (60.0%)	24 (38.7%)	89 (66.4%)	31 (70.5%)	
Severe	29 (12.1%)	8 (12.9%)	14 (10.4%)	7 (15.9%)	
**Skin Injury** – n (%)					
Small	10 (4.2%)	8 (13.1%)	2 (1.5%)	0 (0.0%)	**< 0.001**‡
Large	230 (95.8%)	53 (86.9%)	132 (98.5%)	45 (100.0%)	
**Gustilo type** – n (%)					
IIIA	49 (20.7%)	17 (28.3%)	23 (17.2%)	9 (20.9%)	0.42†
IIIB	116 (48.9%)	25 (41.7%)	68 (50.7%)	23 (53.5%)	
IIIC	72 (30.4%)	18 (30.0%)	43 (32.1%)	11 (25.6%)	
**Bone loss** – n (%)					
None	29 (12.0%)	10 (16.4%)	19 (14.1%)	0 (0.0%)	0.07†
< 1 cm	51 (21.2%)	14 (23.0%)	28 (20.7%)	9 (20.0%)	
> 1 cm	161 (66.8%)	37 (60.7%)	88 (65.2%)	36 (80.0%)	
**Time injury to 1st admission** – [days – n (%)]					
0	241 (98.8%)	63 (98.4%)	134 (99.3%)	44 (97.8%)	0.42‡
≥ 1	3 (1.2%)	1 (1.6%)	1 (0.7%)	1 (2.2%)	
**Length of stay (1st admission)** – [days, median (IQR)]					
	6.5 (3.0-14.3)	5.0 (3.0–13.0)	6.0 (3.0–12.0)	14.0 (8.0–29.0)	< 0.001*
**Total Length of stay** – [days, median (IQR)]					
	25.5 (10.0-49.8)	17.5 (5.3–56.8)	28.0 (11.0–44.0)	37.0 (13.5–76.0)	0.02*
**Readmissions** – Median (IQR)					
	3.0 (2.0–5.0)	2.0 (1.0–5.0)	3.0 (2.0–6.0)	2.0 (1.0–5.0)	0.002*

*One-Way ANOVA

†Pearson’s Chi-Square Test

‡Fisher’s Exact Test

[All % are calculated out of the available data]

During their surgical management, 27.5% of injuries (*n* = 67/243) required at least one type of flap, with rotational flaps (19.3%, *n* = 147/243) being the most common, and 45.3% required bone graft (*n* = 102/225). The mean number of debridement performed per injury was 2.6 throughout the course of the patients’ treatment with the majority (90.6%, *n* = 221/244) performed on the same day. Fixation was typically applied on the same day or the next day for most patients (95.5%, *n* = 232/243), with a median duration of 11.0 months (IQR 5.0–18.0). Around 41.2% (*n* = 100/243) of the patients required two types of fixators during their follow-up. Among those who received a fixator, 48.6% (*n* = 118/243) had a definitive fixator used at first application, and the most employed fixators were monorail fixators (52.7%, *n* = 128/243). The median time until complete soft tissue closure was 31.5 days (IQR 12.0–58.0) (Table 3).

**Table 3:** Surgical management of study patients, 11 May 2018 to 31 October 2020, Gaza, Palestine

Characteristics	All Patients (*N* = 244)	Orthopedic (*N* = 64)	Orthopedic and Plastic (*N* = 135)	Ortho-Plastic (*N* = 45)	*p*-value
**Surgical – throughout the case management**					
**Rotational flap** – n (%)					
Yes	47 (19.3%)	2 (3.2%)	32 (23.7%)	13 (28.9%)	**< 0.001**†
No	196 (80.7%)	61 (96.8%)	103 (76.3%)	32 (71.1%)	
**Free Flap** – n (%)					
Yes	1 (0.4%)	0 (0.0%)	1 (0.7%)	0 (0.0%)	1.00‡
No	242 (99.6%)	63 (100.0%)	134 (99.3%)	45 (100.0%)	
**Fasciocutaneous Flap** – n (%)					
Yes	26 (10.7%)	1 (1.6%)	20 (14.8%)	5 (11.1%)	**0.02**†
No	217 (89.3%)	62 (98.4%)	115 (85.2%)	40 (88.9%)	
**Split-thickness skin graft (STSG)** – n (%)					
Yes	176 (72.4%)	12 (19.0%)	122 (90.4%)	42 (93.3%)	**< 0.001**†
No	67 (27.6%)	51 (81.0%)	13 (9.6%)	3 (6.7%)	
**Debridement** – [n (Mean ± SD)]					
	2.6 ± 1.7	2.2 ± 1.4	3.0 ± 1.9	2.0 ± 1.3	< 0.001*
**Different fixators** – n (%)					
1	128 (52.7%)	29 (46.0%)	76 (56.3%)	23 (51.1%)	0.30‡
2	100 (41.2%)	27 (42.9%)	52 (38.5%)	21 (46.7%)	
3	15 (6.2%)	7 (11.1%)	7 (5.2%)	1 (2.2%)	
**Definitive fixator type** – n (%)					
Monorail fixator	128 (52.7%)	28 (44.4%)	80 (59.3%)	20 (44.4%)	0.08‡
Ilizarov	51 (21.0%)	15 (23.8%)	22 (16.3%)	14 (31.1%)	
Tsf	58 (23.9%)	16 (25.4%)	31 (23.0%)	11 (24.4%)	
Lrs	6 (2.5%)	4 (6.3%)	2 (1.5%)	0 (0.0%)	
**Internal fixation** – n (%)					
Yes	15 (6.2%)	4 (6.3%)	8 (5.9%)	3 (6.7%)	1.00‡
No	228 (93.8%)	59 (93.7%)	127 (94.1%)	42 (93.3%)	
**Definitive fixator at first application** – n (%)					
Yes	118 (48.6%)	26 (41.3%)	71 (52.6%)	21 (46.7%)	0.32†
No	125 (51.4%)	37 (58.7%)	64 (47.4%)	24 (53.3%)	
**Ankle Spanning**^**#**^ – n (%)					
Yes	17 (10.3%)	6 (14.3%)	7 (7.8%)	4 (12.1%)	0.45‡
No	148 (89.7%)	36 (85.7%)	83 (92.2%)	29 (87.9%)	
**Knee Spanning**^**#**^ – n (%)					
Yes	18 (10.9%)	5 (11.9%)	8 (8.9%)	5 (15.2%)	0.50‡
No	147 (89.1%)	37 (88.1%)	82 (91.1%)	28 (84.8%)	
**Bone Graft**^**#**^ – n (%)					
Yes	102 (45.3%)	26 (47.3%)	52 (40.6%)	24 (57.1%)	0.17†
No	123 (54.7%)	29 (52.7%)	76 (59.4%)	18 (42.9%)	
**Duration till 1st bone graft** – [days, median (IQR)] #					
	235.0 (136.0-380.8)	250.0 (149.3-319.8)	235.0 (122.0-399.5)	219.5 (160.0-473.3)	0.42*
**Total number of surgeries** – (mean ± SD)					
	9.6 ± 4.2	8.3 ± 4.1	10.3 ± 4.0	9.6 ± 4.4	0.008*
**Time injury to fixation** – [days, n (%)]					
0	232 (95.5%)	61 (96.8%)	127 (94.1%)	44 (97.8%)	0.53‡
≥ 1	11 (4.5%)	2 (3.2%)	8 (5.9%)	1 (2.2%)	
**Time to definitive stabilization** – [days, n (%))					
Median (IQR)	97.0 (0.0-257.0)	114.0 (0.0-276.0)	57.0 (0.0-259.8)	133.5 (0.0-209.8)	0.06†

IQR interquartile range, SD standard deviation

*One-Way ANOVA

†Pearson’s Chi-Square Test

‡Fisher’s Exact Test

# Ankle and Knee spanning had 79 (32.4%) missing records each; bone graft had 19 (7.8%) missing records; duration till bone graft was missing in 144 (59.0%) of records

[All % are calculated out of the available data]

### Injury burden on patients and healthcare system in Gaza

**Fig. 1:**
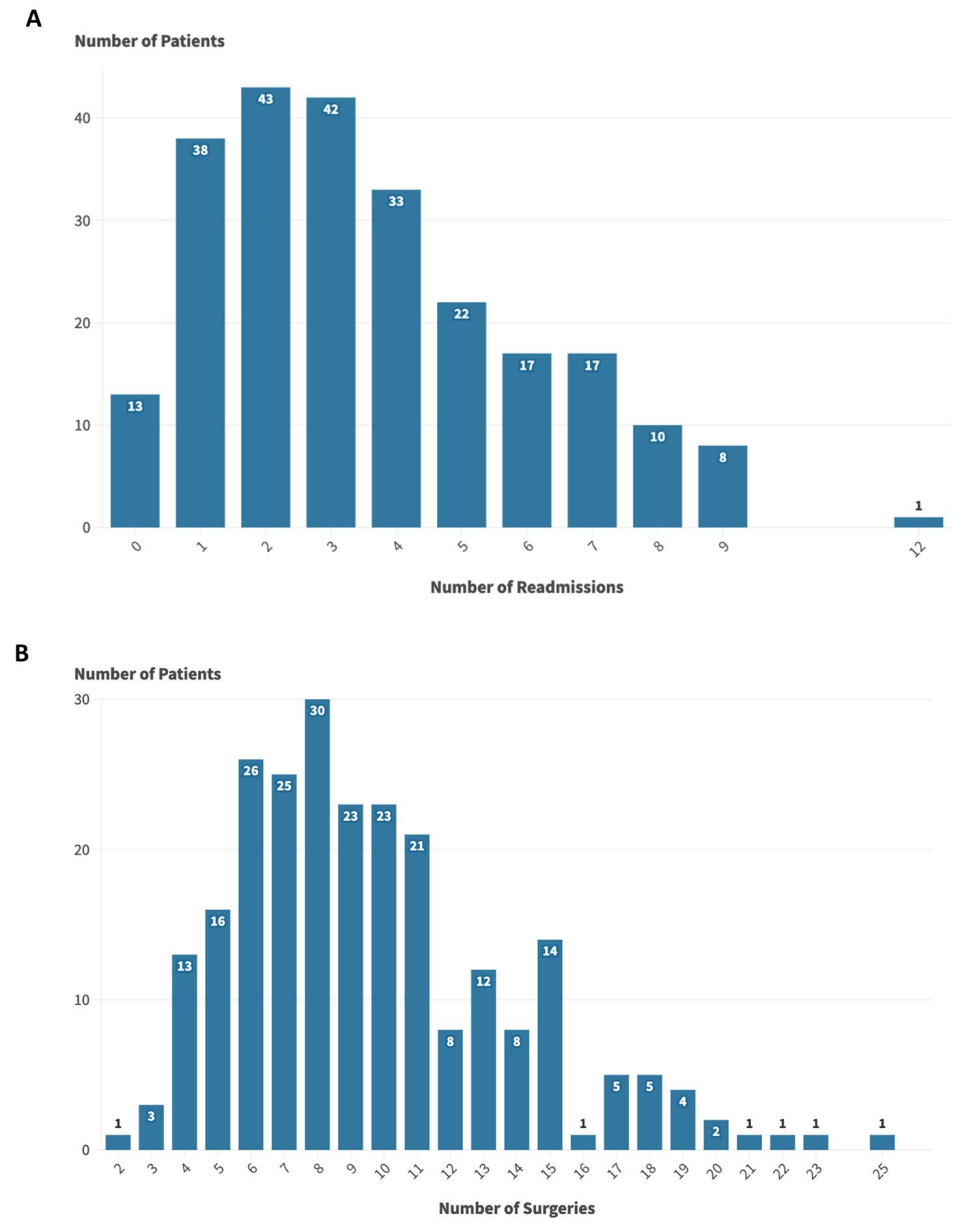
Burden of Injury on Patients and Healthcare System for Open Tibial Fractures in Gaza, Palestine. (**A**) Number of readmissions among patients with open tibial fractures, May-2018 to September-2019, Gaza, Palestine. (**B**) Number of surgeries per patient with open tibial fractures, May-2018 to September-2019, Gaza, Palestine

Notably, a substantial majority of patients (69.3%, *n* = 169/244) had less than 5 readmissions (Fig. 1A). Most patients (58.6%, *n* = 143/244) underwent 5 to 10 surgeries (Fig. 1B). A significant number of patients (53.3%, *n* = 130/244) had a total length of hospital stays of less than 30 days, 41.0% (*n* = 100/244) between 30 and 100 days, and 5.7% (*n* = 14/244) experienced stays over 100 days **(Data not shown)** with a median duration of 25.5 days (IQR 10.0-49.8) (Table 2). Among the 244 patients, open tibial fractures were found on both sides in 8 individuals (3.27%) **(Data not shown)**. Non-union was evident in 53.0% of patients (*n* = 110/234), while delayed amputation occurred in 3.4% of the cases (*n* = 8/244). Infection was observed in 92.5% of the cases (*n* = 172/186) (Table 4).

**Table 4:** Surgical outcomes of study patients, 11 May 2018 to 31 October 2020, Gaza, Palestine

Surgical Outcomes	All Patients (*N* = 244)	Orthopedic (*N* = 64)	Orthopedic and Plastic (*N* = 135)	Ortho-Plastic (*N* = 45)	*p*-value
**Union** – *n* (%)					
Yes	110 (47.0%)	26 (44.1%)	68 (51.9%)	16 (36.4%)	0.18†
No (non-union)	124 (53.0%)	33 (55.9%)	63 (48.1%)	28 (63.6%)	
**Time till union#** – [months, median (IQR)]					
	8.0 (4.0–14.0)	10.0 (5.8–16.0)	6.5 (4.0-14.3)	10.5 (4.8–12.0)	0.34^⋆^
	216.0 (130.0-304.0)	257.0 (162.0-326.0)	212.0 (120.5–349.0)	190.0 (123.0-286.0)	
**Malalignment** – n (%)					
Yes	19 (7.8%)	4 (6.3%)	12 (8.9%)	3 (6.7%)	0.85‡
No	224 (92.2%)	59 (93.7%)	123 (91.1%)	42 (93.3%)	
**Shortening** – n (%)					
Yes	86 (35.4%)	26 (41.3%)	45 (33.3%)	15 (33.3%)	0.53†
No	157 (64.6%)	37 (58.7%)	90 (66.7%)	30 (66.7%)	
**Infection**^**#**^ – n (%)					
Yes	172 (92.5%)	42 (91.3%)	97 (92.4%)	33 (94.3%)	0.87‡
No	14 (7.5%)	4 (8.7%)	8 (7.6%)	2 (5.7%)	
**Pin site Infection** – n (%)					
Yes	168 (69.4%)	41 (65.1%)	94 (70.1%)	33 (73.3%)	0.63†
No	74 (30.6%)	22 (34.9%)	40 (29.9%)	12 (26.7%)	
**Osteomyelitis**^**&**^ – n (%)					
Yes	46 (47.4%)	10 (41.7%)	26 (51.0%)	10 (45.5%)	0.74†
No	51 (52.6%)	14 (58.3%)	25 (49.0%)	12 (54.5%)	
**Delayed Amputation** – n (%)					
Yes	8 (3.4%)	1 (1.6%)	3 (2.3%)	4 (9.1%)	0.08‡
No	227 (96.6%)	60 (98.4%)	127 (97.7%)	40 (90.9%)	
**Time in fixator**^**#**^**–** [months, n (%)]					
< 12	109 (50.9%)	22 (40.7%)	67 (55.8%)	20 (50.0%)	0.26†
≥ 12-<24	71 (33.2%)	23 (42.6%)	37 (30.8%)	11 (27.5%)	
≥ 24	34 (15.9%)	9 (16.7%)	16 (13.3%)	9 (22.5%)	
**Duration till complete soft tissue closure -** [days, Median (IQR)]					
	31.5 (12.0–58.0)	3.0 (0.0-30.8)	36.0 (25.5–70.3)	20.5 (7.3–65.0)	< 0.001*

*One-Way ANOVA

†Pearson’s Chi-Square Test

‡Fisher’s Exact Test

# There were 58 (23.8%) missing records for infection; 30 (12.3%) patients did not have a record for time in fixator; duration till union missing for 116 (47.5%) patients

& No records on osteomyelitis available for 147 (60.2%) patients. When patients with missing osteomyelitis record were all assumed to have had osteomyelitis at some point during their follow-up, the prevalence of osteomyelitis was estimated of 79.1% (*n* = 193), versus 18.9% (*n* = 46) when they were considered as not having had it throughout their follow-up did. No statistical difference was observed between the groups in both cases

[All % are calculated out of the available data]

### Surgical management and outcomes by specialty teams

Orthopedic surgeons treated 64 patients (26.2%), 135 patients (55.3%) had both orthopedic and plastic surgeons involved in their surgical management but not part of a fully integrated team, and ortho-plastic teams treated 45 patients (18.4%). Ortho-plastic teams treated more severe cases of muscle injury (15.9%, *n* = 7/44) compared to the other two groups ([10.4%, *n* = 14/135] for orthopedic and plastic group and [12.9%, *n* = 8/62] for the orthopedic group; *p*-value < 0.001) (Table 2). Additionally, all injuries treated by the ortho-plastic team (*n* = 45/45) were large skin injuries compared to 98.5% (*n* = 132/134) and 86.9% (*n* = 53/61) for the orthopedic and plastic team and the orthopedic only team, respectively (*p*-value < 0.001) (Table 2). Ortho-plastic teams also treated most injuries with a bone loss > 1 cm as well as those with fibula, nerve, or artery and vein injuries (Table 2) and had the lowest median duration till the first bone graft between the groups. However, none of those results were statistically significant (Table 2). Notably, ortho-plastic teams significantly tended to perform less debridement (mean of 2.0 per injury) compared to other groups (3.0 for orthopedic and plastic and 2.2 for orthopedic teams) (Table 3).

Regarding hospitalization, patients treated by an ortho-plastic team spent on average more than double the time at hospital (median of 14.0 days) at their first admission compared to the two other groups (6.0 days for the orthopedic and plastic and 5.0 days for the orthopedic group; *p*-value < 0.001). The average total length of stay for patients treated by ortho-plastic, orthopedic and plastic, and orthopedic teams was 37.0, 28.8, and 17.5 days, respectively (Table 2).

### Surgical outcomes by specialty teams

Of 244 patients, 110 (47.0%) had a bone union within 12 months post-injury while 124 (53.0%) had non-union. The median union time of all patients was 8.0 months. Non-union was more common among patients treated by an ortho-plastic team (63.6%, *n* = 28/44) compared to other groups (*p*-value = 0.18). Median duration till complete soft tissue closure was statistically lower for orthopedic (3.0 days) and ortho-plastic teams (20.5 days) compared to the ortho and plastic group (36.0 days) (*p*-value < 0.001). Other surgical outcomes included bone shortening in 35.4% (*n* = 86/243) of patients, bone mal alignment in 7.8% (*n* = 19/243), and delayed amputation in 3.4% (*n* = 8/235). The highest number of amputations was reported among patients treated by the ortho-plastic team (9.1%, *n* = 4/44) (Table 4).

Infection was observed in 172/186 patients (92.5%). Pin site infection was observed in 168/242 patients (69.4%) and at least 47.4% (*n* = 46/97) of patients had osteomyelitis at some point during their follow-up (when all patients with a missing study record on osteomyelitis were assumed not to have had osteomyelitis during the study period – **data not shown**) (Table 4). Around 40 patients had both osteomyelitis and pin site infection, with 9 treated by both ortho-plastic, 23 by ortho and plastic, and 8 solely by orthopedic teams **(data not shown)**. None of the differences in the bone or infection outcomes among the three groups was statistically significant. Supplementary Fig. 1 illustrates the distribution of outcomes based on the treating teams.

### Predictors of non-union

In logistic regression, patients with bone loss > 1 cm had 17 times higher odds of non-union [aOR 16.62, 95%CI (1.83-150.63)], and those with a vascular injury had 3 times higher odds compared to those without [aOR 2.79, 95%CI (1.17–6.67)]. Placing a definitive fixator on the first application was associated with a lower chance of non-union compared to those who did not have a definitive fixator at the first application [aOR 0.24, 95%CI (0.10–0.56)]. Being treated with an orthopedic and plastic surgeon separately decreased the likelihood of having a non-union, however this was not statistically significant. Although the bivariate analysis showed that those with non-union tend to be older, to smoke, to have more complex injuries (Gustilo type IIIB fracture, an injury involving a fibula or a moderate muscle, undergo more surgical procedures), two different types of fixators throughout their surgical management, be more frequently re-admitted, and stay longer in fixation, those predictors lost their significance in the multivariate model (Supplementary Tables 1 and Table 2.

## Discussion

The substantial burden of GMR injuries on both patients and the healthcare system in the Gaza Strip is evident. Notably, the patients predominantly consisted of young males, reflecting the demographic profile of those affected by violence-related trauma in conflict zones [21]. Around 50% of patients sustained Gustilo IIIB open tibial fractures, accompanied with nerve injuries, large skin injuries, and over 1 cm of bone loss, highlighting the severity and complexity of cases and the need for extensive surgical interventions, such as debridement and soft tissue reconstruction [22]. This suggests that open fractures during conflicts tend to be more severe compared to other LMICs without conflict, where most fractures are of moderate severity (Gustilo I and Gustilo II), accounting for 59% of cases, while severe fractures (Gustilo IIIB and Gustilo IIIC) make up 17% and 5%, respectively [23]. Additionally, the prolonged duration until complete soft tissue closure, the extended hospital stays, and the need for multiple surgeries and admissions, further emphasize the enduring impact and heightened burden of those injuries on the patients and on the already under-resourced and overburdened Gaza healthcare system [24]. We also identified a noteworthy non-union rate among patients (53.0%), which is notably higher than the estimated global range of 10–30% for non-union in open tibial fractures [25–28]. This can be attributed to the challenging healthcare environment in the Gaza Strip, where limited resources, including medical supplies, personnel, and infrastructure, often hinder the delivery of adequate care, especially in emergency situations [24]. However, the comparison of Gaza’s figures with those of other war-affected nations is challenging due to restricted data availability. Also, there appears to be a significant occurrence of infections among these injury cases, which is commonly seen in this injury type in combat casualties [29]. However, our ability to analyze the osteomyelitis prevalence was hampered by data limitations. Furthermore, the occurrence of limb amputation was limited to just 8 patients (3.2%), a figure that falls below the typical range of 10–20% [9–12]. The reported number here might underestimate actual amputation needs due to unsalvageable limbs. Cultural and religious beliefs in the Gaza Strip can cause decision delays, potentially leading to underestimating amputations as an outcome measure [23, 30].

While there was an increasing protective trend in terms of the risk of non-union when treated by ortho-plastic teams compared to either orthopedic or separate orthopedic and plastic teams, this study did not establish a direct correlation between the surgical team type and improved bone outcomes. It is crucial to account for several unexamined factors that might have influenced these results. The absence of significant outcome disparities may stem from sample size limitations and potential triaging favoring ortho-plastic teams for severe or non-healing cases. As a result, patients treated by ortho-plastic teams spent more time hospitalized compared to other teams, increasing the likelihood of developing non-union -as defined per X-ray imaging. Another factor is the difference in the quality of the overall surgical treatment provided by the three different teams, the operating conditions under which the surgical teams were providing care in the different sites, or the type of equipment used and available, all of which would have affected outcomes of care including bone union. In addition, the dissimilarities in clinical and surgical characteristics among the three groups could be influenced, at least partially, by distinct clinical protocols at each site. These variations might encompass divergent discharge criteria and treatment durations. For instance, the observed divergence in hospital stays might not exclusively reflect case severity but may also be influenced by discharge protocols and cost considerations. Patients might choose early discharge, even against medical advice, to minimize out-of-pocket expenses. Notably, this observation is speculative and cannot be definitively verified through the current study’s data. Finally, the experience of orthopedic teams in managing such cases may have also played a role in influencing bone outcomes, particularly in the context of limited availability of plastic surgeons in Gaza [23, 30].

Our results do not negate the potential efficacy of ortho-plastic teams in improving clinical outcomes in this context. Rather, they underscore the multifaceted nature of factors influencing bone recovery, indicating the need for a nuanced exploration of the complexities involved. In fact, there is a significant incidence of morbidity resulting from these injuries and a substantial overall percentage of flap procedures, indicating the necessity of plastic surgery involvement [31]. In addition, gunshot injuries have distinctive characteristics such as openings, compound fragmentation, and bone loss, necessitating meticulous and extensive debridement at an early stage, followed by prompt bone coverage and wound closure. Achieving this might entail providing training to orthopedic surgeons in plastic surgery techniques or fostering collaboration between orthopedic and plastic surgeons. This approach can contribute to improved outcomes and reduce the risk of non-union. Considering the persistent state of emergency, chaos, and intricate nature of injuries observed in the Gaza Strip, it is imperative to prioritize the establishment of a robust infrastructure and allocation of necessary resources to ensure the provision of high-quality care and favorable outcomes. This entails the availability of well-equipped operating theaters, diagnostic facilities, and a reliable electricity supply. Furthermore, access to an ample supply of medical resources, such as dressings, antibiotics, and bone graft materials, is crucial in order to mitigate the risk of complications.

In this study, a number of risk factors that contribute to the increased likelihood of non-union in patients with open tibial fractures were identified. These risk factors include significant bone loss (> 1 cm), vascular injury, and the use of bone graft. The presence of these risk factors is not unexpected, as previous research has established that the severity of injury is a known predictor of non-union [26, 32]. Furthermore, our analysis revealed that the use of definitive fixation is a protective factor against non-union, suggesting that the use of a definitive fixator on the first application may potentially reduce the odds of non-union in patients with open tibial fractures.

### Strengths and limitations

Although this study was first looking into the potential impact of surgical treating teams on outcomes of care in a context like Gaza, it had some limitations. The retrospective design in such a context where data collection is not always a priority and is not standardized affected the completeness of the data. This has potentially impacted on the validity and reliability of the results. However, we were able to mitigate that by using multiple data sources to collect and triangulate the data specifically on main study variables such as the outcome. Missing data instances in this study encompass the relatively small count of reported debridement procedures for this injury type potentially due to their perceived insignificance in medical records in Gaza strip. Additionally, a substantial amount of data is missing on osteomyelitis, which might lead to underestimating its prevalence, as we anticipate it to be higher. Although the cohort of patients included in this study covered a long period as of 2018 and is considerably like other studies around the topic, we believe the sample size was not enough to confidently conclude on risk factors. This can be seen through wide confidence intervals observed in the logistic analysis. However, we believe that it still provided important insights on the complexity of the injuries treated in Gaza but may further limit the operational usability and generalizability of the findings. Despite these limitations, we believe that this study provided valuable information about the characteristics of patients treated by different surgical teams in Gaza and their surgical outcomes and can be used to inform future research.

## Conclusion

This study provides valuable insights into the burden, management, and outcomes of violence-related limb injuries, specifically open tibial fractures caused by gunshots, in the context of the Gaza Strip. The findings highlight the severity and complexity of these injuries. The prolonged duration until complete soft tissue closure and the high number of admissions and surgeries per patient further contribute to the burden on the healthcare system. The non-union rate among patients is notably higher than the global range, indicating the need for improved treatment strategies. The involvement of ortho-plastic teams in the healthcare system of Gaza appears to play a crucial role in managing more severe cases. However, further research is still needed to understand how to better manage those injuries.

### Electronic Supplementary Material

Below is the link to the electronic supplementary material.

Supplementary Material 1

## Data Availability

All data relevant to the study are included in the article or uploaded as supplementary information.

## Declarations

### Patient and public involvement

Patients and/or the public were not involved in the design, conduct, reporting or dissemination plans of this research.

## Abbreviations

ERB: Ethics Review Board
GMR: Great March of Return
GSW: Gunshot wounds
HIC: High-Income Country
IQR: Interquartile range
IRB: Institutional Review Board
LMICs: Low- and Middle-Income Countries
MAP: Medical Aid for Palestinians
MoH: Ministry of Health
MSF: Médecins Sans Frontières
SD: Standard deviation
STSG: Split-thickness skin graft
UHWC: Union of Health Work Committees
