# Supplementary material for: The integration of ortho-plastic limb salvage teams in the humanitarian response to violence-related open tibial fractures: evaluating outcomes in the Gaza Strip
Source: Confl Health. 2024 Apr 24;18:35. doi: 10.1186/s13031-024-00596-3 (PMC11040898; doi:10.1186/s13031-024-00596-3)
Supplement: Supplementary file 1 — Supplementary Material 1 [file 13031_2024_596_MOESM1_ESM.docx]

**Supplementary Data**


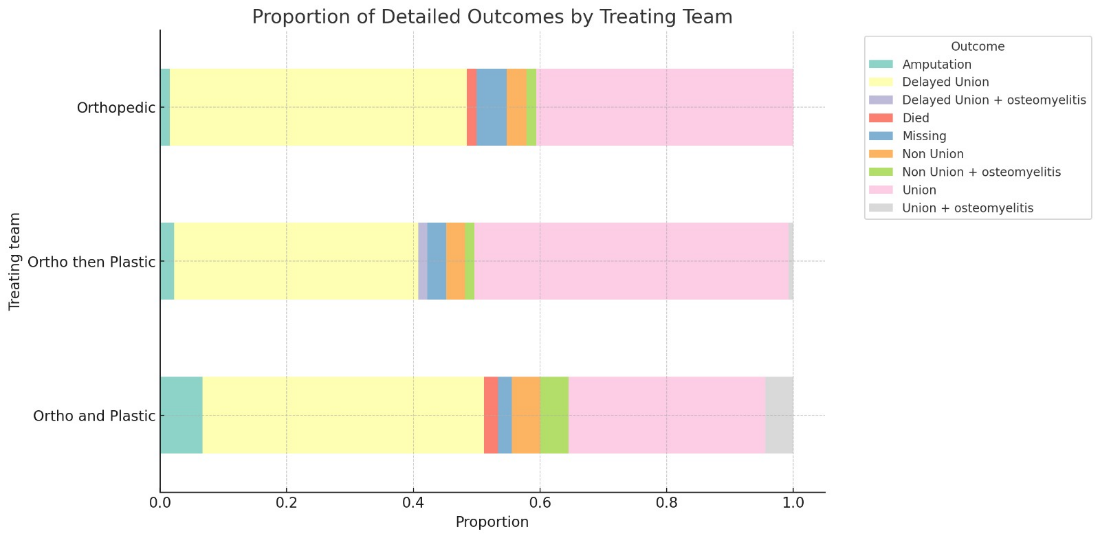


**Supplementary Figure 1.** Proportion of detailed outcomes by treating team.

**Supplementary Table 1.** Predictors of non-union among patients with gunshot open tibial fractures, 11 May 2018 to 31 October 2020, Gaza, Palestine

|  | **Union**  (N=110) | **Non-union**  (N=124) | **OR (95% CI)** | **aOR (95% CI)** | **p-value** |
| --- | --- | --- | --- | --- | --- |
| **Treating team** – n (%) |  |  |  |  |  |
| Orthopedic | 26 (23.6%) | 33 (26.6%) | 1 | 1 |  |
| Ortho then Plastic | 68 (61.8%) | 63 (50.8%) | 0.73 (0.39, 1.35) | 0.68 (0.25, 1.85) | 0.45 |
| Ortho and Plastic | 16 (14.5%) | 28 (22.6%) | 1.38 (0.62, 3.07) | 0.79 (0.22, 2.79) | 0.71 |
| **Amount of bone loss (at presentation)** – n (%) | | | | |  |
| No bone loss | 25 (22.7%) | 2 (1.6%) | 1 | 1 |  |
| Minimal (1 cm) | 38 (34.5%) | 12 (9.7%) | 3.95 (0.81, 19.16) | 8.98 (0.87, 92.45) | 0.06 |
| Significant bone loss (> 1cm) | 47 (42.7%) | 110 (88.7%) | 29.26 (6.66, 128.54) | 16.62 (1.83, 150.63) | **0.01** |
| **Vascular Injury** – n (%) |  |  |  |  |  |
| No | 78 (70.9%) | 57 (46.0%) | 1 | 1 |  |
| Yes | 32 (29.1%) | 67 (54.0%) | 2.87 (1.67, 4.93) | 2.79 (1.17, 6.67) | **0.02** |
| **Fixator used at 1st application was definitive** (No) | 81 (73.6%) | 32 (25.8%) | 0.12 (0.07, 0.22) | 0.24 (0.10, 0.56) | **0.001** |
| **Bone Graft** (No) | 4 (3.8%) | 97 (81.5%) | 48.06 (20.76, 111.27) | 37.48 (14.14, 99.34) | **<0.001** |

Patients with missing records on their union outcome (n=10) were excluded from the analysis.

1. Variables imposed in the model: Treating team.

2. Variables selected based on significance: Age, Smoking, Gustilo type III, Amount of bone loss (at presentation), Site of injury, Nerve Injury, Vascular Injury, Muscle Injury, Skin, Number of debridement, Time to fixation from injury in days, NSAIDs, Fibula involved, Rotational flap, Nb of different Types of fixators, Definitive fixator type, Fixator used at 1st application was definitive, Bone Graft, Duration till complete soft tissue closure in days, Pin site Infection, Amputation, LOS 1st admission (days), Number of readmissions, Total number of surgeries, Total LOS in all admissions in Days, Date from stabilization to definitive (days).

3. Variables excluded because of missing values > 5%: Infection, Time in fixator (months), Duration till union in months.

**Supplementary Table 2.**  Associations between all Characteristics and Union vs. Non-Union.

| **Characteristics, n (%)** | **Union**  **N = 110**  **(47.0%)** | **Non-Union**  **N = 124**  **(53.0%)** | **p-value** |
| --- | --- | --- | --- |
| **Age, years** – Mean ± SD | 27.5 ± 7.8 | 30.1 ± 8.5 | **0.01**** |
| **Gender** |  |  |  |
| Male | 110 (100.0%) | 122 (98.4%) | 0.50‡ |
| Female | 0 (0.0%) | 2 (1.6%) |  |
| **Smoking** |  |  |  |
| Yes | 45 (41.3%) | 66 (54.5%) | 0.04† |
| No | 64 (58.7%) | 55 (45.5%) |  |
| **Gustilo type III** |  |  |  |
| a | 32 (29.1%) | 17 (14.0%) | **0.01**† |
| b | 51 (46.4%) | 60 (49.6%) |  |
| c | 27 (24.5%) | 44 (36.4%) |  |
| **Amount of bone loss (at presentation)** | | | |
| No bone loss | 25 (22.7%) | 2 (1.6%) | **<0.001**† |
| Minimal (1 cm) | 38 (34.5%) | 12 (9.7%) |  |
| Significant bone loss (> 1cm) | 47 (42.7%) | 110 (88.7%) |  |
| **Site of injury** |  |  |  |
| Proximal | 32 (29.1%) | 38 (30.6%) | 0.95† |
| Mid | 45 (40.9%) | 51 (41.1%) |  |
| Distal | 33 (30.0%) | 35 (28.2%) |  |
| **Fibula involved** |  |  |  |
| Yes | 51 (46.4%) | 79 (63.7%) | **0.01**† |
| No | 59 (53.6%) | 45 (36.3%) |  |
| **Nerve Injury** |  |  |  |
| Yes | 79 (71.8%) | 95 (77.2%) | 0.34† |
| No | 31 (28.2%) | 28 (22.8%) |  |
| **Vascular Type injured Combined** |  |  |  |
| No vascular injury | 78 (70.9%) | 57 (46.0%) | **<0.001**† |
| Artery OR Vein injury | 19 (17.3%) | 32 (25.8%) |  |
| **Muscle Injury** |  |  |  |
| Mild | 44 (40.0%) | 21 (17.1%) | **<0.001**† |
| Moderate | 59 (53.6%) | 81 (65.9%) |  |
| Severe | 7 (6.4%) | 21 (17.1%) |  |
| **Ankle Spanning** |  |  |  |
| Yes | 6 (7.6%) | 10 (11.8%) | 0.37† |
| No | 73 (92.4%) | 75 (88.2%) |  |
| **Knee Spanning** |  |  |  |
| Yes | 7 (8.9%) | 11 (12.9%) | 0.40† |
| No | 72 (91.1%) | 74 (87.1%) |  |
| **Skin Injury** |  |  |  |
| Small | 5 (4.6%) | 3 (2.4%) | 0.36‡ |
| Large | 103 (95.4%) | 121 (97.6%) |  |
| **Rotational flap** |  |  |  |
| Yes | 17 (15.5%) | 28 (22.6%) | 0.17† |
| No | 93 (84.5%) | 96 (77.4%) |  |
| **Free Flap** |  |  |  |
| Yes | 1 (0.9%) | 0 (0.0%) | 0.47‡ |
| No | 109 (99.1%) | 124 (100.0%) |  |
| **Fasciocutaneous Flap** |  |  |  |
| Yes | 11 (10.0%) | 13 (10.5%) | 0.90† |
| No | 99 (90.0%) | 111 (89.5%) |  |
| **Split-thickness skin graft (STSG)** |  |  |  |
| Yes | 76 (69.1%) | 95 (76.6%) | 0.20† |
| No | 34 (30.9%) | 29 (23.4%) |  |
| **Number of debridement** |  |  |  |
| Median (IQR) | 2.0 (1.0-3.0) | 2.0 (2.0-3.0) | 0.45** |
| **Number of different Types of fixators** | | | |
| 1 | 84 (76.4%) | 37 (29.8%) | **<0.001**† |
| 2 | 25 (22.7%) | 73 (58.9%) |  |
| 3 | 1 (0.9%) | 14 (11.3%) |  |
| **Definitive fixator type** |  |  |  |
| Monorail fixator | 81 (73.6%) | 40 (32.3%) | **<0.001**‡ |
| Ilizarov | 14 (12.7%) | 37 (29.8%) |  |
| tsf | 14 (12.7%) | 42 (33.9%) |  |
| lrs | 1 (0.9%) | 5 (4.0%) |  |
| **Internal fixation** |  |  |  |
| Yes | 4 (3.6%) | 9 (7.3%) | 0.23† |
| No | 106 (96.4%) | 115 (92.7%) |  |
| **Fixator used at 1st application was definitive** | | | |
| Yes | 81 (73.6%) | 32 (25.8%) | **<0.001**† |
| No | 29 (26.4%) | 92 (74.2%) |  |
| **Bone Graft** |  |  |  |
| Yes | 4 (3.8%) | 97 (81.5%) | **<0.001**† |
| No | 100 (96.2%) | 22 (18.5%) |  |
| **Infection** |  |  |  |
| Yes | 73 (97.3%) | 98 (89.9%) | 0.05† |
| No | 2 (2.7%) | 11 (10.1%) |  |
| **Wound healing** |  |  |  |
| Yes | 107 (99.1%) | 120 (99.2%) | 1.00‡ |
| No | 1 (0.9%) | 1 (0.8%) |  |
| **Amputation** |  |  |  |
| Yes | 1 (0.9%) | 6 (4.9%) | 0.13‡ |
| No | 106 (99.1%) | 117 (95.1%) |  |
| **Time from injury to 1st admission** |  |  |  |
| 0 | 110 (100.0%) | 122 (98.4%) | 0.50‡ |
| ≥1 | 0 (0.0%) | 2 (1.6%) |  |
| **LOS 1st admission (days)** | | | |
| Median (IQR) | 4.5 (2.3-12.0) | 9.0 (5.0-21.0) | **<0.001***** |
| **Number of readmissions** |  |  |  |
| Median (IQR) | 3.0 (1.0-4.0) | 4.0 (2.3-6.0) | **<0.001***** |
| **Total number of surgeries** |  |  |  |
| Median (IQR) | 7.0 (6.0-10.0) | 11.0 (9.0-14.0) | **<0.001***** |
| **Total LOS in all admissions in Days** |  |  |  |
| Median (IQR) | 16.0 (6.0-30.3) | 41.5 (19.0-73.3) | **<0.001***** |
| **Time to fixation from injury in days** |  |  |  |
| Median (IQR) | 0.0 (0.0 – 0.0) | 0.0 (0.0 – 0.0) | 0.65** |
| **Date from stabilization to definitive** – days | |  |  |
| Median (IQR) | 0.0 (0.0-63.3) | 207.0 (97.0-377.0) | **<0.001**** |
| **Time in fixator** – months |  |  |  |
| Median (IQR) | 6.0 (4.0-11.0) | 16.0 (11.0-24.0) | **<0.001**** |
| **Duration till union** – months |  |  |  |
| Median (IQR) | 5.0 (4.0-9.0) | 17.0 (12.0-24.3) | **<0.001**** |
| **Treating team** |  |  |  |
| Orthopedic | 26 (23.6%) | 33 (26.6%) |  |
| Ortho then plastic | 68 (61.8%) | 63 (50.8%) | **0.18†** |
| Ortho and plastic | 16 (14.5%) | 28 (22.6%) | **0.18†** |

**Independent Samples T-Test

†Pearson’s Chi-Square Test

‡Fisher’s Exact Test

Outcome of All Patients (N = 234, the 10 patients missing information were excluded from the data analysis)

[All % are calculated out of the available data]
